# Surfactant-tuned vanadium pentoxide for enhanced photocatalytic degradation of organic dyes: nanosheet *vs.* microflower morphologies†

**DOI:** 10.1039/d5ra01050k

**Published:** 2025-05-22

**Authors:** Minh Nguyet Thi Quan, Duy Van Lai, Matteo Tonezzer, Dat Quang Do, Duc D. La

**Affiliations:** a School of Engineering Physics, Hanoi University of Science and Technology (HUST) No 1 Dai Co Viet Street Hanoi City Vietnam; b Institute of Materials Science, Academy of Science and Technology 18 Hoang Quoc Viet Street, Cau Giay District Hanoi City Vietnam; c Department of Food Quality and Nutrition, Research and Innovation Centre, Fondazione Edmund Mach San Michele all’ Adige, TN 38010 Italy; d Department of Chemical and Geological Sciences, University of Cagliari Campus of Monserrato (CA) I09042 Monserrato Italy; e School of Materials Science and Engineering (SMSE), Hanoi University of Science and, Technology (HUST) No 1, Dai Co Viet Street Hanoi Vietnam; f Department of Natural Sciences, Hoa Lu University Ninh Binh City Vietnam; g Laboratory of Biophysics, Institute for Advanced Study in Technology, Ton Duc Thang University Ho Chi Minh City Vietnam laducduong@tdtu.edu.vn; h Faculty of Applied Sciences, Ton Duc Thang University Ho Chi Minh City Vietnam

## Abstract

This study explores the impact of surfactants on the morphology and photocatalytic performance of V_2_O_5_ materials synthesized *via* a hydrothermal method, followed by calcination. Two surfactants, Pluronic P123 and cetrimonium bromide (CTAB), were used, leading to the formation of V_2_O_5_ nanosheets (V_2_O_5_-P123) and V_2_O_5_ microflowers (V_2_O_5_-CTAB), each exhibiting distinct structural characteristics and photocatalytic efficiencies. The prepared nanosheets are of sizes ranging from a few micrometers and thickness of approximately 50–60, and the flower-like structures have an average diameter of approximately 5 micrometers. The photocatalytic degradation of Methylene Blue (MB) and Rhodamine B (RhB) dyes were evaluated under simulated sunlight. The V_2_O_5_-P123 nanosheets showed superior performance, achieving 96.72% MB degradation in 150 minutes, with a degradation rate constant of 0.92 min^−1^, compared to 90.02% for V_2_O_5_-CTAB microflowers (0.51 min^−1^). This enhanced performance was attributed to the larger surface area, higher porosity, and optimized structure of the nanosheets, which promoted better dye adsorption and interaction with reactants. In contrast, the degradation of RhB was lower for both structures, with the V_2_O_5_-P123 nanosheets achieving only 18% degradation (rate constant: 0.0014 min^−1^) and the V_2_O_5_-CTAB microflowers 46% (rate constant: 0.0071 min^−1^). This highlights the challenge of degrading RhB due to its complex molecular structure and higher chemical stability, limiting effective interaction with the catalyst surface. The study emphasizes the significant influence of surfactants on the morphology and photocatalytic performance of V_2_O_5_ materials, with V_2_O_5_ nanosheets showing promising potential for environmental remediation, particularly in the degradation of organic pollutants like MB. The findings also suggest that optimizing the photocatalyst design could enhance the degradation of more chemically stable pollutants, thereby expanding the scope of applications for clean energy production and environmental protection.

## Abstract 2

This study explores surfactants' impact on V_2_O_5_ morphology and photocatalytic performance. V_2_O_5_ nanosheets (V_2_O_5_-P123) exhibited better MB degradation (96.72%) than microflowers (90.02%) due to a larger surface area and optimized structure.

## Graphical abstract

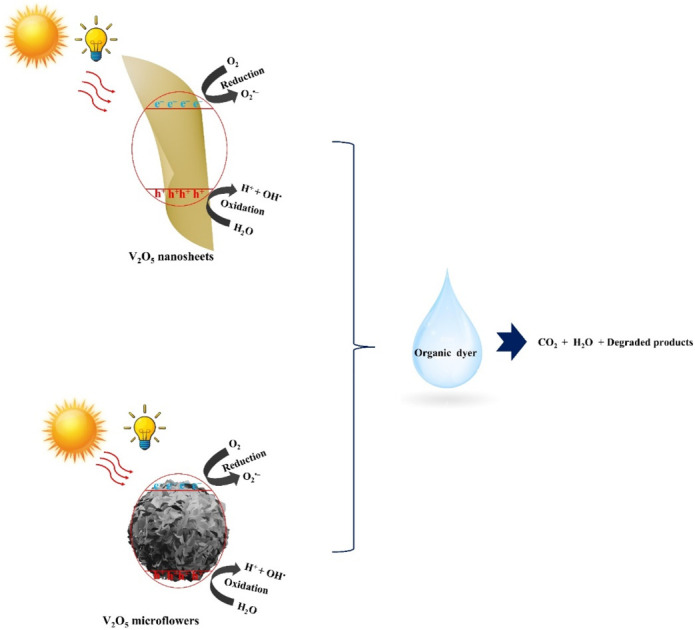

## Introduction

The rapid growth of population and industrialization has led to excessive fossil fuel consumption, resulting in severe environmental pollution, irreversible climate change, and an urgent need for sustainable solutions.^1^ Water pollution caused by organic and inorganic compounds, particularly industrial dyes and agricultural pesticides, poses significant threats to the environment and human health. This necessitates immediate attention and solutions to safeguard clean water resources.^2,3^

Although conventional wastewater treatment methods are effective for many pollutants, they often face limitations in efficiently removing organic dyes, highlighting the need to explore advanced techniques.^4^ Advanced Oxidation Processes (AOPs) are modern water treatment methods that utilize hydroxyl radicals (OH˙) to effectively degrade complex organic compounds, including persistent dyes like methylene blue, into less toxic products. Among these, photocatalysis has emerged as a sustainable and efficient solution for industrial wastewater treatment and mitigating the global energy crisis due to its ability to harness renewable and inexhaustible solar energy.^5,6^ Notably, semiconductor-based photocatalysis is recognized as an effective strategy for solar energy conversion.^7^ Photocatalytic semiconductors play a crucial role in photocatalytic technology. Among them, metal oxides like V_2_O_5_ are highly valued for their efficient visible light absorption (bandgap ∼ 2.3 eV), chemical stability, low cost, abundant availability, high solar spectrum absorption efficiency, non-toxic nature, and biocompatibility. Compared to other semiconductors such as ZnO, ZrO_2_, TiO_2_, SnO_2_, VO_*x*_, MoO_*x*_, metal sulfides (CdS), or organic compounds, V_2_O_5_ overcomes durability limitations and shows promising potential for applications in lithium-ion batteries, solar cells, gas sensors, catalysts, and optoelectronics.^5,8–11^

Photocatalytic performance depends on the semiconductor's ability to respond to light, which is closely related to its intrinsic properties, such as morphology, size, and energy band structure. To meet the multifunctional requirements of photocatalysts, recent studies have developed V_2_O_5_-based catalysts with diverse morphologies and structures using various synthesis methods, including hydrothermal synthesis,^12^ co-precipitation,^13^ solvothermal method,^14^ and chemical exfoliation.^15^ Selecting a suitable synthesis method, characterized by simplicity, environmental friendliness, and cost-effectiveness, plays a critical role in tailoring the structure and morphology of the catalyst.^16^ One of the most commonly used methods for preparing V_2_O_5_, especially for photocatalytic applications, is the hydrothermal method.

The hydrothermal method enables the control of V_2_O_5_ material morphology, allowing the formation of structures ranging from 0D nanoballs and nanoflowers to 1D nanowires and nanorods, and 2D nanosheets, by adjusting factors such as solvent, acid concentration, pH, precursor concentration, and surfactant type.^17^ 2D materials, characterized by their large specific surface area, strong light absorption, and enhanced density of active states due to structural defects, have demonstrated exceptional performance in photochemical reactions, particularly in the rapid degradation of organic dyes. The synergy of superior optical and surface properties, combined with high stability and recyclability, positions 2D materials as leading candidates for environmental remediation and renewable energy applications.^18–20^

The hydrothermal synthesis duration significantly influences morphology, surface area, porosity, and crystallinity of the materials, thereby affecting their photocatalytic efficiency. Optimizing the reaction time can improve crystallinity and reduce defects; however, excessive reaction times may lead to particle agglomeration, reducing the surface area and catalytic performance. For example, Rasheed *et al.*^21^ demonstrated that the presence of CTAB as a surfactant during synthesis resulted in the formation of thinner nanoflake particles with an average diameter of approximately 70 nm after annealing at 500 °C for 2 hours. Similarly, Abdullah *et al.*^22^ observed that extending the annealing duration to 4 hours at the same temperature produced nanoflakes with slightly increased thicknesses of 65–80 nm. Mao Tang *et al.*^23^ revealed that hydrothermal time significantly influences TiO_2_'s crystalline phase, morphology, and photocatalytic performance, with TiO_2_-12 h (anatase phase, 145.3 m^2^ g^−1^ surface area) achieving superior degradation of rhodamine B (99.6%) and tetracycline hydrochloride (90.0%) due to its high surface area and efficient charge separation. However, the study lacks an exploration of long-term stability, broader pollutant applicability, and optimization of mixed-phase performance, as well as detailed mechanistic insights into photocatalytic pathways. Qing Su and colleagues fabricated V_2_O_5_/TiO_2_ heterostructured nanomaterials with various morphologies using a one-step electrospinning process. They investigated the effects of annealing temperature and the V/Ti molar ratio on the visible-light photocatalytic performance, as demonstrated by the photocatalytic degradation of Rhodamine B (RhB).^24^

In the study by Islam Ibrahim and colleagues,^25^ the addition of different surfactants (SDS, Tween 80, Triton X-100, and PVA) during the synthesis of V_2_O_5_ photocatalysts significantly influenced the materials' morphology, crystallinity, and optoelectronic properties. Among these, Tween 80 (T80) exhibited the best photocatalytic performance, reducing hexavalent chromium and degrading methylene blue and tetracycline, suggesting its potential as an efficient bifunctional photocatalyst for both oxidation and reduction processes. However, there is a gap in the comparison of surfactants based on their polarity and nature in the context of V_2_O_5_ synthesis, an area that remains underexplored, particularly for non-ionic surfactants like Tween 80.

This study investigates the role of surfactants in shaping the morphology and photocatalytic performance of V_2_O_5_ materials. Pluronic P123 and CTAB were employed in a hydrothermal synthesis process, resulting in V_2_O_5_ nanosheets (V_2_O_5_-P123) and nanoflowers (V_2_O_5_-CTAB) with distinct morphological and catalytic properties. The findings revealed that V_2_O_5_-P123 achieved a superior Methylene Blue (MB) degradation efficiency of 96.72% after 150 minutes, attributed to its larger surface area and higher porosity, compared to 90.02% for V_2_O_5_-CTAB. However, both materials exhibited lower performance for Rhodamine B (RhB) degradation, highlighting the impact of RhB's complex molecular structure on catalytic activity. This study emphasizes the significant influence of surfactants on the morphology and functionality of V_2_O_5_ and suggests its potential application in organic pollutant remediation, paving the way for the development of more efficient photocatalytic materials.

## Experimental section

### Chemicals

All chemicals used for the synthesis of V_2_O_5_ nanosheets and V_2_O_5_ microflowers were of analytical grade and sourced from Sigma-Aldrich (St. Louis, MO, USA). These included ammonium metavanadate (NH_4_VO_3_, ≥98%), Pluronic P-123 (poly(ethylene glycol)-*block*-poly(propylene glycol)-*block*-poly(ethylene glycol), HO(CH_2_CH_2_O)_20_(CH_2_CH(CH_3_)O)_70_(CH_2_CH_2_O)_2_0H, 99%), hexadecyltrimethylammonium bromide (CTAB, ≥98%), oxalic acid (H_2_C_2_O_4_, ≥99%), ethylene glycol (C_2_H_6_O_2_, ≥99%), and ethanol (CH_3_CH_2_OH, 99.8%). Deionized (DI) water was used as the solvent for all preparation and testing processes.

### Synthesis of V_2_O_5_ nanosheets

V_2_O_5_ nanosheets were synthesized by hydrothermal method, in which ammonium metavanadate was used as precursor and deionized water acted as solvent, following the procedure described in previous studies.^26^

In this process, 10 mmol of NH_4_VO_3_ is dissolved in a mixture of 30 mL of deionized water and 30 mL of ethylene glycol and stirred for 15 minutes. Pluronic P-123 is then added, and the mixture is stirred for an additional 30 minutes. Next, 10 mmol of oxalic acid is added, the pH is adjusted to 4, and the solution is stirred for another 15 minutes. The resulting solution is transferred to a 100 mL Teflon reaction vessel and subjected to hydrothermal treatment at 200 °C for 24 hours. After cooling, the precipitate is collected by centrifugation, washed several times with deionized water, and then twice with ethanol. Finally, the product is dried at 60 °C for 24 hours and calcined at 500 °C for 2 hours.

### Synthesis of V_2_O_5_ nanoflowers

V_2_O_5_ microflowers were synthesized by hydrothermal method, using ammonium metavanadate (NH_4_VO_3_) as precursor. The synthesis procedure was optimized based on previous studies^10,26^ using a solvent mixture of ethylene glycol (C_2_H_6_O_2_) and deionized water in a 1 : 1 ratio. During this process, the surfactant hexadecyltrimethylammonium bromide (CTAB) was added to regulate the formation of microflower structure.

Initially, 1.2 grams of NH_4_VO_3_ and 2 grams of hexadecyltrimethylammonium bromide (CTAB) were dissolved in 40 mL of deionized water and 40 mL of ethylene glycol. The solution was stirred for 30 minutes to ensure complete dissolution of the reagents. The pH of the solution was then adjusted to 4 by adding 1.26 grams of oxalic acid (H_2_C_2_O_4_), followed by an additional 15 minutes of stirring.

The resulting mixture was transferred into a 100 mL Teflon-lined stainless steel autoclave and subjected to hydrothermal treatment at 200 °C for 24 hours. After natural cooling to room temperature, the solution was centrifuged at 4000 rpm to collect the precipitate. The obtained precipitate was washed several times with deionized water and ethanol to eliminate impurities. Finally, the product was dried in an oven at 60 °C for 24 hours and subsequently calcined at 500 °C for 2 hours to obtain the final V_2_O_5_ nanoflower material.

### Material characterization

The morphological, microstructural, and compositional characteristics of the synthesized V_2_O_5_ nanosheets and V_2_O_5_ microflowers were analyzed using a variety of techniques. Field emission scanning electron microscopy (FE-SEM, JEOL 7600F) and high-resolution transmission electron microscopy (HR-TEM, JEM 2100, JEOL Ltd) were employed to investigate the surface morphology and crystal structure. The phase structure of the materials was determined by powder X-ray diffraction (XRD, Advance D8, Bruker) using Cu-Kα_1_ radiation (*λ* = 1.54056 Å). Elemental composition was confirmed through energy dispersive X-ray spectroscopy (EDS, 7395H, Horiba), while molecular structure characteristics were examined by Raman spectroscopy (Renishaw, InVia resonance micro-Raman spectrometer).

Additionally, the specific surface area (S_BET) and pore size distribution of the nanomaterials were determined through nitrogen adsorption/desorption measurements at 77 K, utilizing a Micromeritics Gemini VII 2390 instrument. Optical absorption spectra were recorded using a UV-vis spectrophotometer (Cary 100 Conc, Varian). The photocatalytic performance of the V_2_O_5_ sample was evaluated by testing the degradation of methylene blue (MB) dye, with the results analyzed using UV-vis spectroscopy.

### Photocatalytic experiments

The photocatalytic performance of V_2_O_5_ nanosheets and V_2_O_5_ microflowers in the degradation of Rhodamine B (RhB) and Methylene Blue (MB) was evaluated at room temperature under visible light using a 350 W air-cooled xenon lamp source (China). In the experiment, 20 mL of RhB solution with an initial concentration of 10 mg L^−1^ was mixed with 0.03 g of photocatalyst and stirred continuously in the dark at room temperature for 60 min to reach adsorption–desorption equilibrium. Then, the solution was irradiated with visible light from a xenon lamp placed 15 cm away from the sample. At specified time intervals, about 3 mL of the solution was taken out, centrifuged to remove the catalyst, and UV-vis absorption spectra were recorded to analyze the degradation of the pollutants. Real-time UV-vis spectra were measured at the characteristic wavelengths of RhB (*λ*_max_ = 553 nm) and MB (*λ*_max_ = 664 nm), thereby evaluating the photocatalytic efficiency of V_2_O_5_ materials in the degradation of RhB and MB.

## Results and discussion

### Morphological and microstructural characterization

**Fig. 1:**
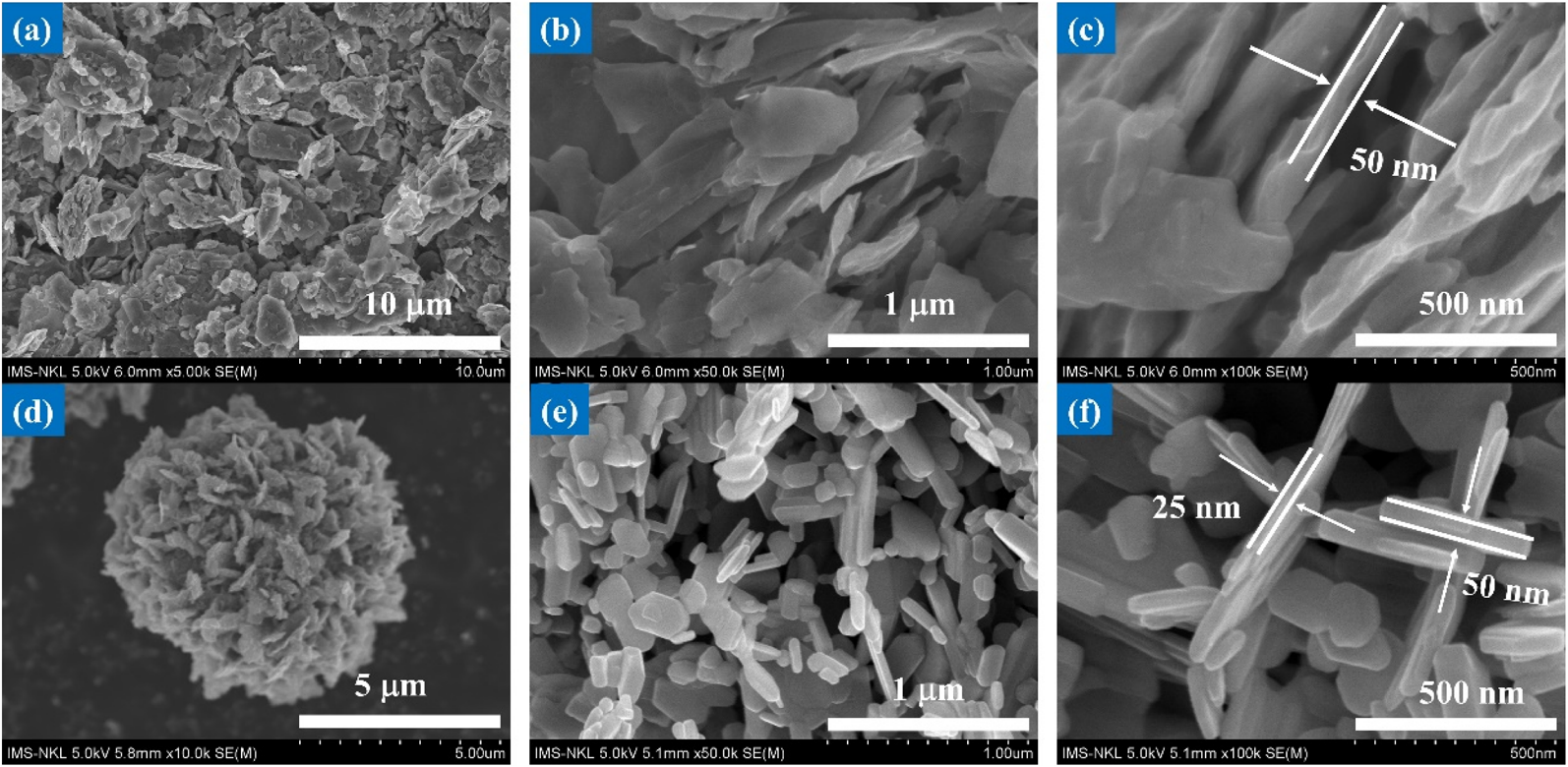
FE-SEM images at low and high magnifications of different V_2_O_5_ morphologies: (a)–(c) nanosheets and (d)–(f) flower-like structures composed of nanosheets, obtained after annealing at 500 °C.

FE-SEM images at low and medium magnifications of V_2_O_5_ nanosheets and microflowers are presented in Fig. 1. The nanosheets, with sizes ranging from a few micrometers, are clearly observed (Fig. 1(a–c)). Notably, the edges of the nanosheets appear with a certain roughness, which makes them different from the previous flat sheets. This morphology is reminiscent of the layered structure of materials such as graphene oxide (rGO) and molybdenum disulfide (MoS_2_), which are characterized by tight coupling between nanosheets.^27^

Nanosheets exhibit remarkable width and thinness, arranged meticulously in parallel orientation. The high-magnification image in Fig. 1(b) and (c) indicates an average thickness of approximately 50–60 (nm). This morphology is attributed to variations in growth rates along different crystal orientations, resulting in a significant surface area. This nanosheet-shaped structure facilitates the transport of the electrolyte across the catalyst surface. It makes active surface area increase for redox reactions, consequently, enhancing the effectiveness of photocatalytic degradation.

During the synthesis of nanomaterials, solvents and surfactants play a crucial role in controlling their structure and morphology.^26,28^ When ethylene glycol (C_2_H_6_O_2_) was used as the solvent in combination with the surfactant hexadecyltrimethylammonium bromide (CTAB), relatively spherical structures were formed. This contrasts significantly with the two-dimensional structures obtained when water (H_2_O) was used as the solvent along with the Pluronic P-123 surfactant.^26^

**Fig. 2:**
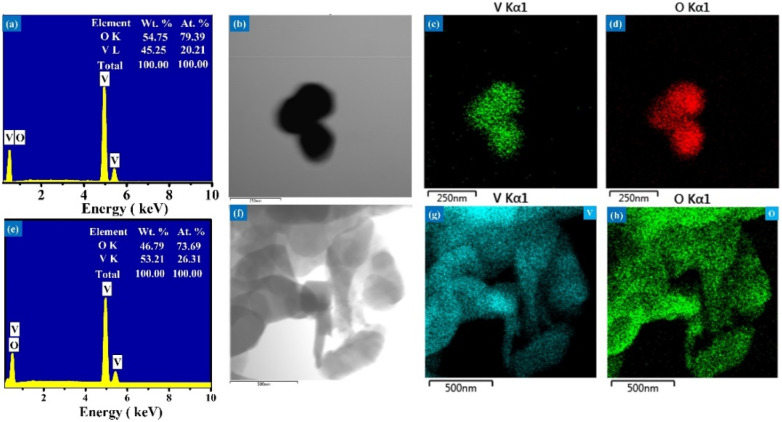
Elemental composition and distribution of V_2_O_5_ nanosheets and microflowers (a) and (e) EDS spectra showing the elemental composition of V_2_O_5_ nanosheets and microflowers, respectively. (b)–(d), (f)–(h) Elemental mapping images displaying the uniform distribution of vanadium (V) and oxygen (O) in the V_2_O_5_ nanosheets (b)–(d) and microflowers (f)–(h).

Fig. 2(d–f) presents low and medium magnification FESEM images that illustrate the formation of flower-like spherical structures of V_2_O_5_, synthesized in ethylene glycol and deionized water in the presence of oxalic acid (H_2_C_2_O_4_). The SEM images reveal a uniform distribution of microflowers, each assembled from thin, densely packed nanosheets. This demonstrates the precise control achieved during the synthesis process, enabling the formation of structures with uniformly sized sheets.

These flower-like structures have an average diameter of approximately 5 micrometers, demonstrating the ability to control size at the micrometer scale. However, the thickness of the petals is only a few nanometers, resulting in a large surface area, which is advantageous for applications requiring high surface area, such as catalysis, energy storage, and gas sensing.

The formation of spherical microflowers V_2_O_5_ from thin nanosheets under hydrothermal conditions can be explained by the transformation of ammonium metavanadate (NH_4_VO_3_) according to the reaction:

1 2NH_4_VO_3_ → V_2_O_5_·*x*H_2_O + 2NH_4_^+^ + (1 − *x*)H_2_O

This reaction results in the formation of V_2_O_5_·*x*H_2_O, with the thin nanosheets self-assembling to create V_2_O_5_ microflowers structures.

The elemental compositions of the V_2_O_5_ nanosheet and microflower samples were determined using energy dispersive X-ray spectroscopy (EDS), with the results presented in Fig. 2(a) and (e). Both samples consisted solely of vanadium (V) and oxygen (O) elements, with no peaks associated with impurities or contaminants. The atomic percentages of vanadium and oxygen in the V_2_O_5_ nanosheet sample were 20.21% and 79.39%, respectively, while the V_2_O_5_ microflower sample had 26.31% and 73.69%, respectively. These values reflect an approximate 2 : 5 ratio between vanadium and oxygen, which is consistent with the chemical formula of V_2_O_5_. This result confirms the successful synthesis and purity of the V_2_O_5_ structures studied. The elemental maps of vanadium and oxygen in the V_2_O_5_ nanosheets and microflowers (Fig. 2b–d and f–h) reveal a uniform distribution of these elements across all V_2_O_5_ materials.

**Fig. 3:**
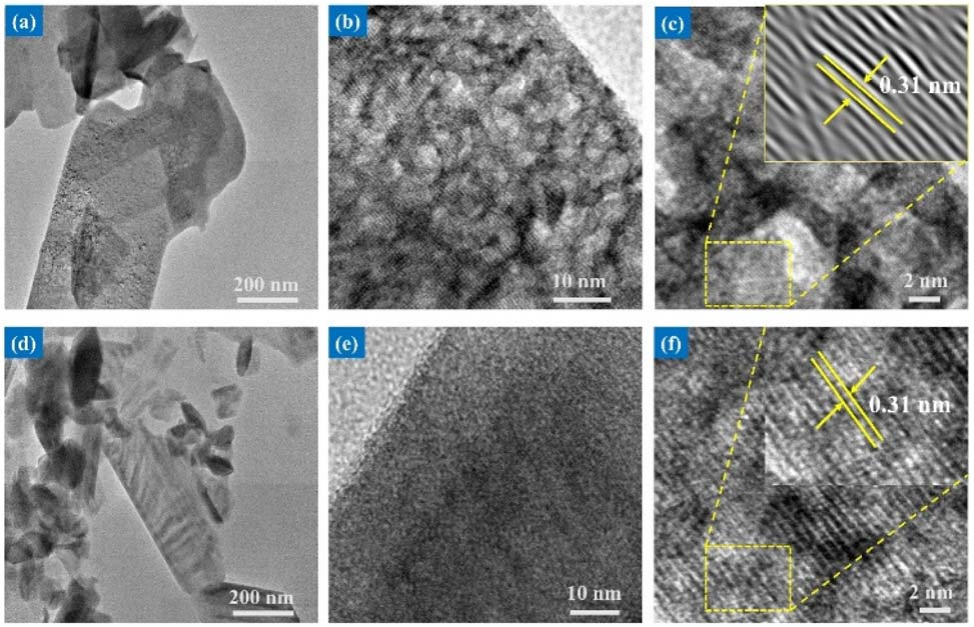
TEM, HRTEM micrograph, and lattice interplanar of different V_2_O_5_ morphologies: (a)–(c) nanosheets, and (d)–(f) micro-flowers.

The high-resolution TEM technique was employed to gain a deeper understanding of the morphology and crystallography of the V_2_O_5_ nanostructures. As depicted in Fig. 3(a–c), the synthesized V_2_O_5_ nanosheets exhibit a size range of several hundred nanometers, with a porous structure indicative of excellent crystalline quality, which is beneficial for enhancing material properties and performance. The HRTEM images in Fig. 3(b) and (c) show the uniform distribution of pores and the nanocrystalline nature of the V_2_O_5_ nanosheets, with each sheet consisting of pores in the range of 4–6 nm. The micro-flowers assembled using CTAB as a template consist of ultra-thin petals with smoother surfaces and lower porosity compared to the nanosheet structures.

The corresponding FFT (Fast Fourier Transform) and interplanar spacing (inset in Fig. 3(c) and (f)) were analyzed using Digital Micrograph software. The observed lattice fringes exhibit a spacing of 0.31 nm, corresponding to the (110) planes of V_2_O_5_, confirming the high crystallinity of the material.^29^ This finding is consistent with the XRD results, reinforcing the notion that the V_2_O_5_ structures are polycrystalline. The high crystallinity of the V_2_O_5_ nanomaterials facilitates charge carrier transport, promoting enhanced material properties and performance.

The influence of surfactants on the material structure formation is crucial. Pluronic P123 facilitates crystal growth along two directions, limiting the final particle size, while CTAB, with its amphiphilic nature, supports the assembly of VO_3_^−^ ions into 3D microspheres.^30^ Modifying the morphological characteristics significantly alters the photocatalytic properties, as the high surface area and porosity enable increased adsorption sites. Thus, the TEM and SAED images provide crucial insights into the structural and compositional characteristics of the V_2_O_5_ materials, highlighting their potential for improved photocatalytic applications.

**Fig. 4:**
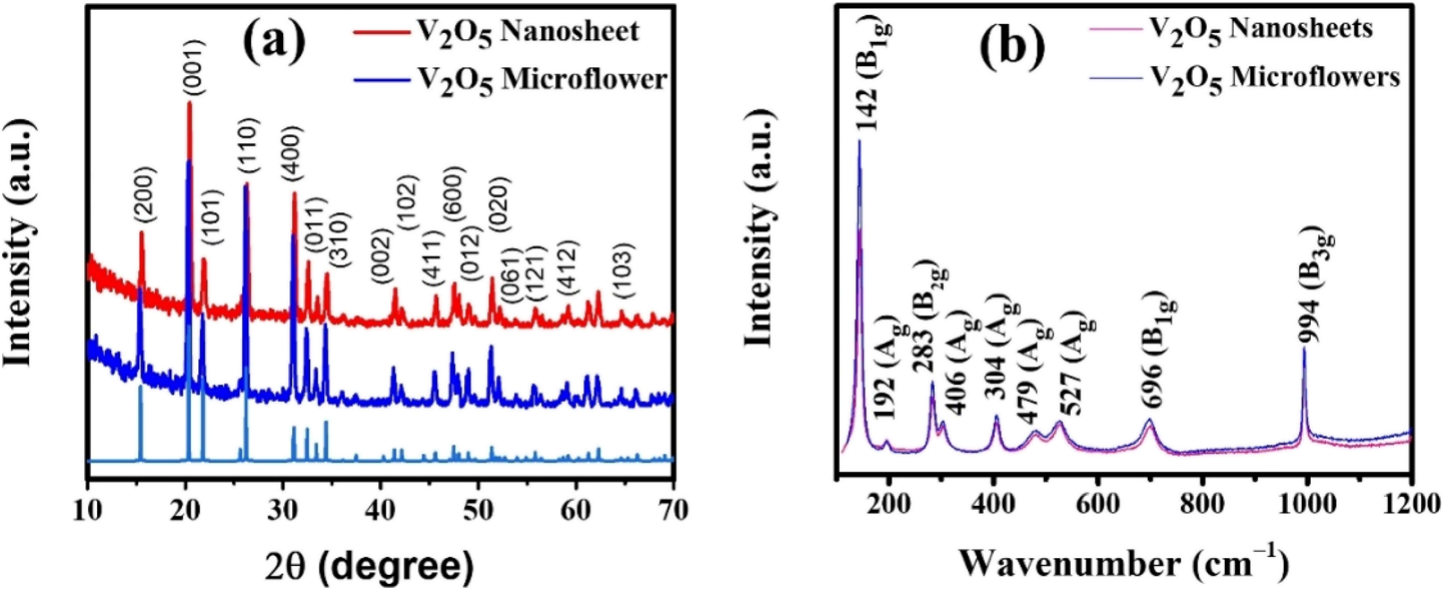
Microstructural characterization of different V_2_O_5_ morphologies: (a) X-ray diffraction patterns, (b) Raman spectroscopy analysis.

Fig. 4(a) displays the XRD patterns of V_2_O_5_ nanosheets and microflowers, confirming their orthorhombic crystal structure (space group: *Pmmn*, JCPDS no. 41-1426).^31,32^ The absence of extraneous peaks indicates high phase purity and crystallinity, with no detectable impurities or secondary phases. The diffraction peaks for both nanosheets and microflowers correspond well to the lattice planes (*e.g.*, (001), (101), (110)), underscoring the structural integrity of these morphologies. Using the Scherrer equation, the crystallite sizes of the nanosheets and microflowers were calculated as 24.09 nm and 26.99 nm, respectively. The smaller crystallite size of the nanosheets suggests a higher surface-to-volume ratio, which likely enhances electron transfer efficiency by reducing the electron transfer distance.^33^

Fig. 4(b) presents the Raman spectra of two samples, V_2_O_5_ nanosheets (red line) and microflowers (blue line), after annealing at 500 °C, illustrating the characteristic features of the orthorhombic α-V_2_O_5_ crystal structure. The Raman spectrum of the nanosheets exhibits sharp peaks at 143, 196, 283, 406, 479, 526, 698, and 994 cm^−1^. The most intense peak at 143 cm^−1^ corresponds to the V–O–V vibration, confirming the distinctive layered structure of the material. The prominent peak at 994 cm^−1^, associated with the stretching vibration of the V=O bond, underscores the high crystallinity and structural order of the nanosheets after annealing. In comparison, the Raman spectra of the microflowers also display peaks characteristic of the orthorhombic structure. However, the broader nature of these peaks suggests the presence of nanoscale effects. Peaks at 144, 284, and 406 cm^−1^ correspond to bending vibrations of V=O bonds, while the peaks at 994 cm^−1^ and 698 cm^−1^ are attributed to the in-phase and out-of-phase stretching vibrations of V=O bonds, respectively. The broadening of the spectral peaks indicates a more complex structure and a distribution of crystallite sizes in the microflowers, consistent with their three-dimensional morphology and pronounced surface effects. A comparison between the two morphologies reveals that nanosheets possess superior crystalline properties, as evidenced by their sharper peaks, reflecting a highly ordered and homogeneous structure. In contrast, microflowers exhibit a more intricate three-dimensional structure with varied bonding and vibrational characteristics. Annealing at 500 °C significantly enhanced the crystal quality of both morphologies, with the microflowers undergoing a more pronounced transformation. These distinct structural features suggest different potential applications: nanosheets are better suited for applications requiring large surface areas and high structural order, while microflowers are advantageous for catalysis and adsorption due to their three-dimensional morphology and nanoscale effects.

**Fig. 5:**
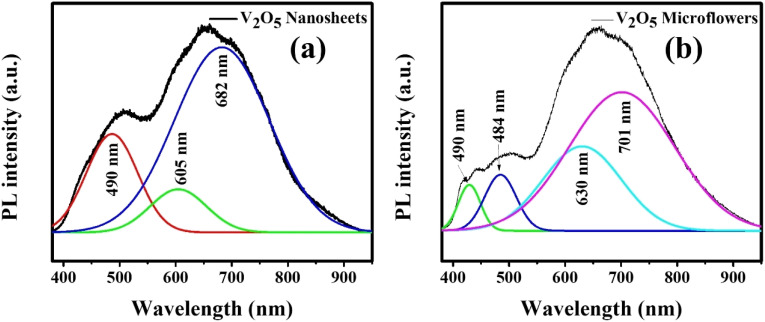
PL spectrum data of V_2_O_5_ nanosheets (a) and microflowers (b) at room temperature (excitation: 355 nm).

The PL spectra of V_2_O_5_ nanosheet and microflower structures measured at room temperature, as shown in Fig. 5, reveal distinct emission peaks. The peak at 490 nm (2.5 eV) corresponds to the band-edge transition, while the peak at 605 nm (2.05 eV) is attributed to direct electronic transitions between O 2p and V 3d states. Additionally, the peak at 630 nm (1.89 eV) is associated with oxygen defect states formed during growth. Notably, V_2_O_5_ nanosheets exhibit stronger PL intensity in the range of 640–710 nm (1.93–1.74 eV) due to interfacial gap states caused by oxygen vacancies, while the intensity is lower in V_2_O_5_ microflowers (Fig. S1 (ESI†)).^8,34,35^ These results highlight the influence of nanostructure morphology on the optical properties of V_2_O_5_

**Fig. 6:**
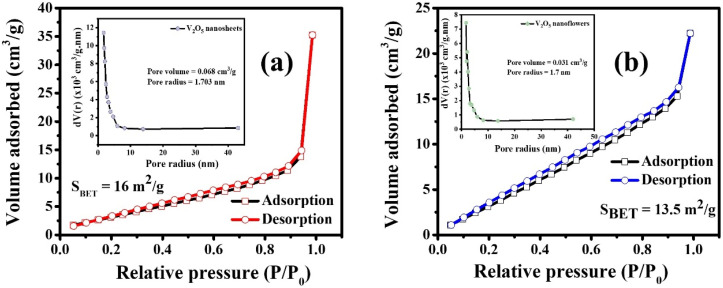
Nitrogen adsorption/desorption isotherms of (a) nanosheet and (b) micro-flower samples annealed at 500 °C. Inset: pore size distribution of each nanomaterial.

Fig. 6 presents the nitrogen adsorption isotherms of V_2_O_5_ samples with different morphologies, including nanosheets and micro-flower structures, annealed at 500 °C. These isotherms are classified as type IV with an H3 hysteresis loop, indicating the presence of mesopores, consistent with a narrow pore size distribution predominantly below 10 nm, as determined by BJH pore size analysis.^36^ Notably, the V_2_O_5_ nanosheet structure shows a larger specific surface area compared to the micro-flower structure, with values of 16 m^2^ g^−1^ and 13.5 m^2^ g^−1^, respectively. The high surface area of the nanosheet structure is crucial for providing more dye adsorption sites and offering additional reaction sites, which significantly enhances the efficiency of photocatalytic degradation.^37^ Furthermore, the pore volume, especially in the nanosheet sample with a value of 0.07 cm^3^ g^−1^, contributes to faster adsorption and degradation of methylene blue dye, thereby improving the photocatalytic yield.

**Fig. 7:**
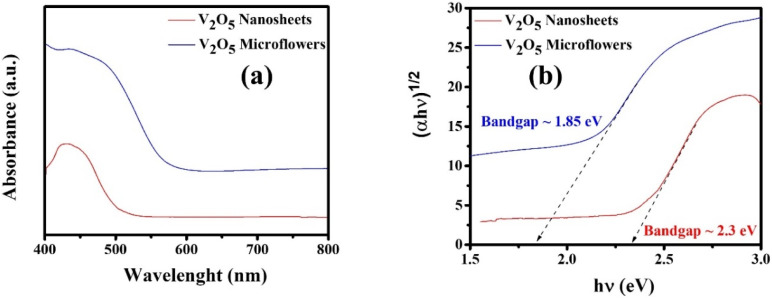
UV-vis spectra of V_2_O_5_ nanosheets and microflowers samples (a), Tauc plots and estimated band gaps of the synthesized materials (b).

Fig. 7(a) shows the UV-vis spectra of V_2_O_5_-CTAB and V_2_O_5_-P123 samples, demonstrating strong absorption in the ultraviolet region for both materials. The observed differences in absorption intensity and spectral shifts highlight the role of surfactants (CTAB and P123) in controlling the morphology and optical properties of the synthesized materials. Fig. 7(b) presents the corresponding Tauc plots, which were used to estimate the bandgap energy *E*_g_ based on the Tauc equation:^38^

1 (*αhν*)^*n*^ = *A*(*hν* − *E*_g_)

where *n* = 1/2 for direct electronic transitions, *A* is a material-dependent constant, and hν is the photon energy calculated from the wavelength *λ* as *hν* = 1239.7/*λ*.

The Kubelka–Munk function *F*(*R*_∞_), derived from diffuse reflectance spectra, replaces the absorption coefficient *α* as follows:^39^

2 *F*(*R*_∞_) = (1 − *R*_∞_)^2^/2*R*_∞_

The bandgap energy *E*_g_ is determined from the plot of (*hνF*(*R*_∞_))^2^*versus hν*, where the tangent at the inflection point intersects the horizontal axis.

The results reveal that the bandgap energy of the V_2_O_5_-CTAB sample is 1.85 eV, while that of the V_2_O_5_-P123 sample is 2.3 eV. This discrepancy can be attributed to the influence of surfactants on the morphology and microstructure of the materials. Specifically, CTAB promotes the formation of surface defects and intermediate states within the bandgap, resulting in a reduction of *E*_g_. Moreover, the smaller particle size and higher porosity observed in the V_2_O_5_-CTAB sample contribute to further narrowing of the bandgap. In contrast, P123 leads to more crystalline structures with fewer surface defects, thereby maintaining a wider bandgap. Thus, surfactants play a significant role in tuning the bandgap energy of materials by influencing morphology, defect density, and surface structure.^40,41^

**Fig. 8:**
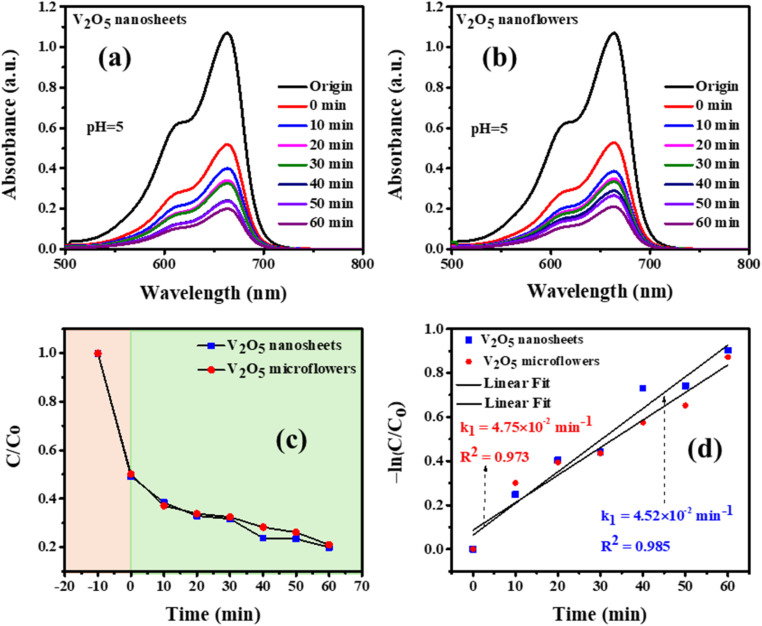
Photocatalytic degradation of Methylene Blue (MB) under simulated sunlight using V_2_O_5_ nanosheets and microflowers: (a) and (b) absorbance spectra over irradiation time, (c) photodegradation efficiency, and (d) ln(*C*/*C*_0_) *vs.* irradiation time.

The photocatalytic performance of V_2_O_5_ nanosheets and microflowers was assessed through their ability to degrade Methylene Blue (MB) and Rhodamine B (RhB) dyes under simulated sunlight, revealing distinct differences between the two morphologies (Fig. 8(a) and (b)). Among the tested samples, V_2_O_5_ microflowers exhibited the highest adsorption capacity after 60 minutes in the dark (Fig. 8(c)). However, V_2_O_5_ nanosheets demonstrated superior removal efficiency, achieving a removal rate of 80.06% for MB, compared to 79.05% for the microflowers (Fig. 8(d)) after 60 minutes. The corresponding rate constants were 0.92 min^−1^ and 0.51 min^−1^, respectively. The nanosheets' enhanced performance can be attributed to their large specific surface area, thin morphology, and orderly alignment, which collectively improve contact with dye molecules, facilitate efficient reactant diffusion, and optimize light absorption. In contrast, the microflowers' more intricate structure likely limits active site accessibility, reducing their catalytic efficiency. After irradiation time increase to 150 minutes witnesses the MB degradation efficiencies up 96.72% and 90.02% by the V_2_O_5_ nanosheets and microflowers, respectively (Fig. S2†).

The decomposition efficiency of the MB dye is determined *via* equation eqn (3).3
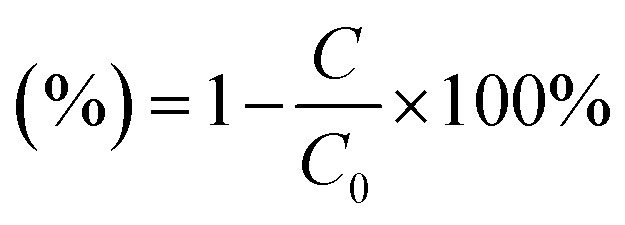
*C*_0_, and *C* denote the initial concentration and the concentration of the MB solution at time *t*. The photocatalytic degradation process follows first-order decay kinetics, as depicted pictorially in 6 (B–C). Additionally, the rate constant in min^−1^ for decay can be determined using equation eqn (3):^42,43^4
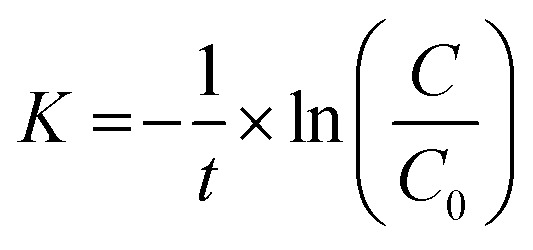
where *C*_0_ and *C* represent the initial concentration and concentration at time *t* of the dye solution in mol L^−1^, respectively, meanwhile, *t* is the irradiation time.

For RhB degradation, both morphologies exhibited significantly lower performance, with degradation efficiencies of 46% for nanosheets and 18% for microflowers after 150 minutes (Fig. 3 (ESI†)). The rate constants for RhB degradation were 0.0071 min^−1^ and 0.0014 min^−1^, respectively. This reduced activity is likely due to the complex molecular structure and higher chemical stability of RhB, which hinder its adsorption onto the catalyst surface. In comparison, the superior degradation of MB suggests that the structural and surface characteristics of V_2_O_5_ provide better interaction with simpler dye molecules.

These findings underscore the potential of V_2_O_5_ nanosheets as highly efficient photocatalysts for MB degradation, emphasizing the importance of structural and surface optimization. However, the lower efficiency for RhB highlights the critical influence of dye molecular properties on photocatalytic outcomes. This study offers valuable guidance for tailoring V_2_O_5_-based photocatalysts for diverse organic pollutants, particularly those with more complex structures.

**Fig. 9:**
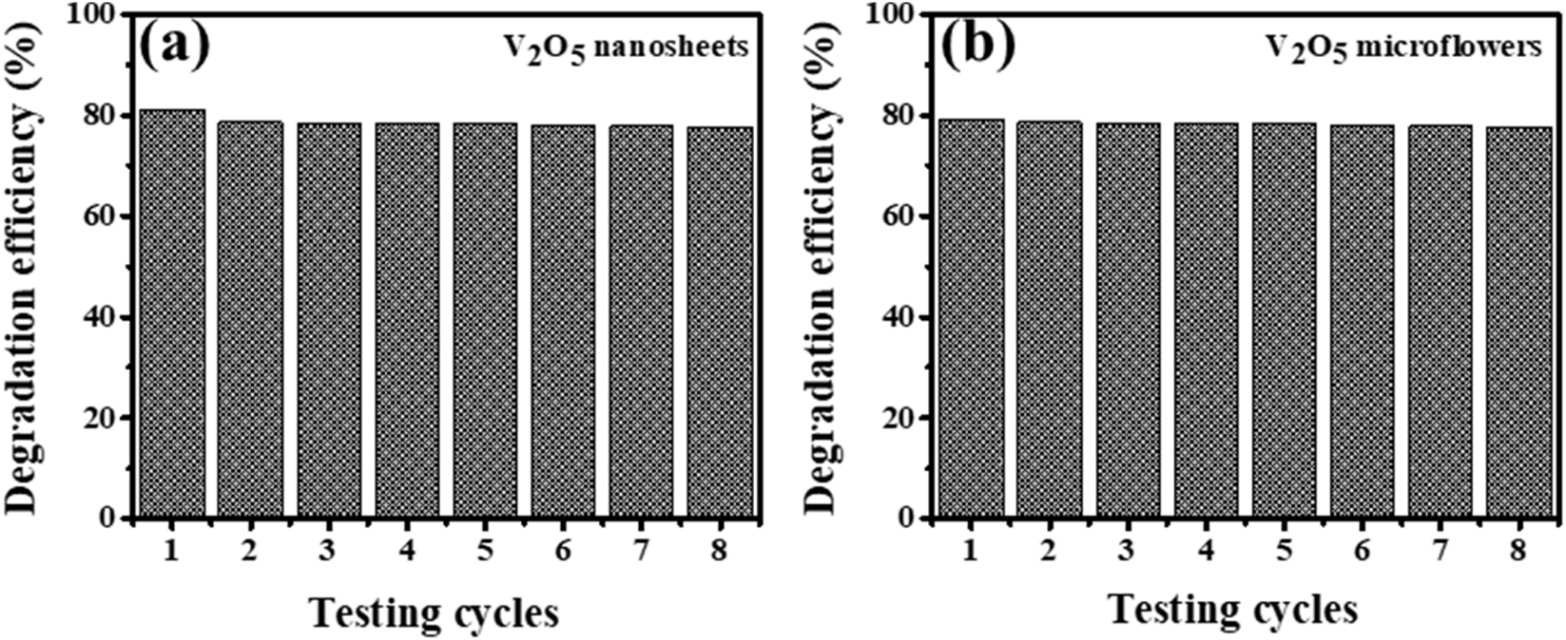
The stability of (a) V_2_O_5_ nanosheets and (b) microflowers photocatalytic activity for the degradation of MB after 60 minutes of light irradiation.

Fig. 9 illustrates the recyclability and stability of V_2_O_5_ nanosheets and microflowers as photocatalysts in the degradation of Methylene Blue (MB) under simulated sunlight over eight consecutive cycles. The MB removal efficiency by V_2_O_5_ nanosheets decreased slightly from 80% in the first cycle to 78.76% in the eighth cycle, corresponding to a reduction of approximately 1.1%. The MB removal efficiency by V_2_O_5_ flowers was also decreased by about 1%. These results demonstrate that the photocatalytic activity of V_2_O_5_ nanosheets and microflowers remains highly effective even after repeated use, highlighting the remarkable stability of this material. This property is particularly significant for practical applications, where durability and recyclability are critical factors. Furthermore, the ease of separation and reuse of the material adds further value to its potential for environmental remediation, especially in removing organic pollutants like dyes. The findings not only affirm the high performance and robustness of V_2_O_5_ nanosheets but also underscore their feasibility for large-scale wastewater treatment systems.

**Fig. 10:**
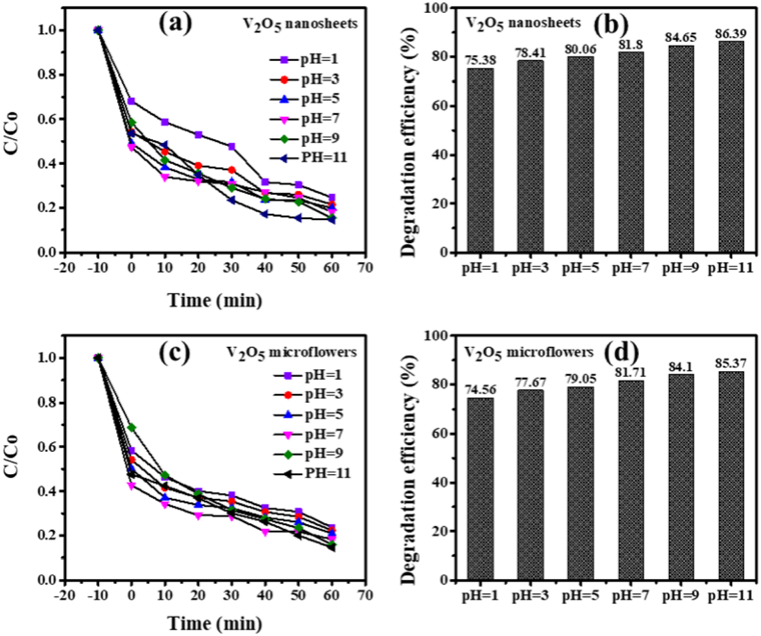
The effect of pH solutions on the photodegradation of MB by (a) and (b) V_2_O_5_ nanosheets and (c) and (d) microflowers photocatalysts after 60 minutes of light irradiation.

The Fig. 10 illustrates the impact of pH on the photocatalytic degradation of Methylene Blue (MB) by V_2_O_5_ nanosheets and microflowers. Both catalysts show faster degradation rates under acidic conditions (pH 1), with the *C*/*C*_0_ ratio decreasing significantly over time, indicating higher photocatalytic efficiency. As pH increases, the degradation rate slows down, with higher pH (pH 9 and pH 11) showing noticeably reduced performance. This trend is attributed to the electrostatic interaction between MB and the catalyst: at acidic pH, V_2_O_5_ acquires a positive charge, enhancing MB adsorption, while at higher pH, the catalyst's negative charge reduces adsorption. Degradation efficiency is highest at pH 11 for both nanosheets (86.39%) and microflowers (85.37%), suggesting a favorable balance between MB adsorption and ROS generation. Overall, the results highlight that acidic conditions favor V_2_O_5_ photocatalysts, but they also demonstrate the material's capability to function across a range of pH levels.

The data presented in Table 1 provides a comprehensive comparison of photocatalytic performance across various V_2_O_5_-based systems, highlighting the exceptional results achieved by V_2_O_5_ nanosheets under simulated sunlight. Achieving a degradation efficiency of 96.72% within 150 minutes, the V_2_O_5_ nanosheets outperform many other systems, including V_2_O_5_-rGO (90%) and V_2_O_5_–Fe_3_O_4_/rGO (76%) under visible light. This superior performance can be attributed to their ultrathin structure, which offers a high surface area and abundant active sites for adsorption and reaction. Although the reaction time is longer compared to Cu-doped V_2_O_5_ (90 minutes), the V_2_O_5_ nanosheets benefit from excellent stability and sustained high efficiency under sunlight, making them more practical for real-world applications. Moreover, the use of a xenon lamp to simulate sunlight highlights their potential for outdoor environmental remediation, surpassing systems designed primarily for visible light. Compared to hybrid materials like V_2_O_5_-rGO, the pristine nanosheets demonstrate a simpler yet highly effective structure, achieving superior results without additional dopants or supports. Their performance also exceeds that of V_2_O_5_ microflowers (90.02%), emphasizing the advantage of nanosheets in providing uniform and accessible active sites. In conclusion, the findings underline the remarkable photocatalytic efficiency of V_2_O_5_ nanosheets and their suitability for practical applications. The results not only confirm their effectiveness in pollutant degradation but also suggest that further optimization of reaction kinetics could enhance their competitiveness compared to other advanced systems.

**Table 1:** Comparative analysis of photodegradation of Methylene Blue (MB) dye using various photocatalysts in comparison with literature

Photocatalyst	Weight of catalyst	Volume MB of the solution	Initial MB solution concentration	Degradation time (min)	Light source	Photodegradation efficiency (%)	Ref.
V_2_O_5_	2 mg	10 mL	10 mg L^−1^	90	White light (100 mW cm^−2^)	68	44
rGO-V_2_O_5_	10 mg	50 mL	50 mg L^−1^	255	Mercury-lamp	85	45
Be-V_2_O_5_	20 mg	10 mL	10 mg L^−1^	90	Visible light	73–88	46
V_2_O_5_-rGO	6 mg	300 mL	3 mg L^−1^	20	Visible light	71	47
V_2_O_5_-rGO	50 mg	20 mL	20 mg L^−1^	50	Visible light	90	48
V_2_O_5_-Fe_3_O_4_/rGO	30 mg	30 mL	9.38 × 10^−6^ M	110	Visible light	76	49
V_2_O_5_ nanosheets	30 mg	20 mL	10 mg L^−1^	150	Sunlight (xenon lamp, 350 W)	96.72	This work
V_2_O_5_ microflowers						90.02	

**Fig. 11:**
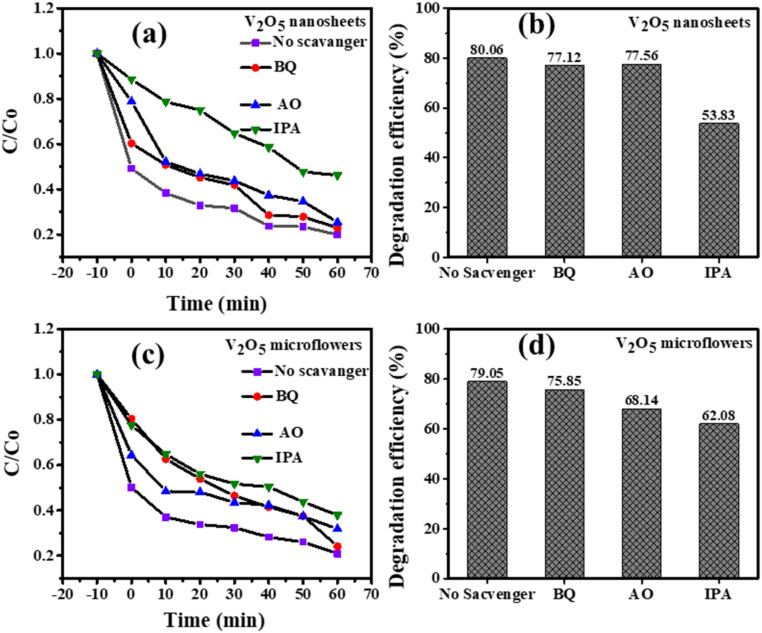
The effect of radical scavengers on the photodegradation of MB by (a) and (b) V_2_O_5_ nanosheets and (c) and (d) microflowers photocatalysts after 60 minutes of light irradiation.

Illustrated in Fig. 11 is the impact of radical scavengers on the photocatalytic degradation of Methylene Blue (MB) using V_2_O_5_ nanosheets and microflowers. Panel (a) and (c) show that the degradation rate of MB is slower in the presence of radical scavengers such as BQ (Benzoquinone), AO (Ammonium Oxalate), and IPA (Isopropyl Alcohol), with IPA exhibiting the strongest inhibition. This indicates that hydroxyl radicals (˙OH), which are scavenged by IPA, play a critical role in the photocatalytic process. Panel (b) and (d) display the degradation efficiency at 60 minutes, with the highest efficiency observed when no scavenger is used (80.06% for nanosheets and 79.05% for microflowers). IPA significantly reduces degradation, highlighting the importance of hydroxyl radicals. Overall, the data suggest that both V_2_O_5_ nanosheets and microflowers are effective photocatalysts, with nanosheets slightly outperforming microflowers, likely due to their higher surface area and more active sites.

**Fig. 12:**
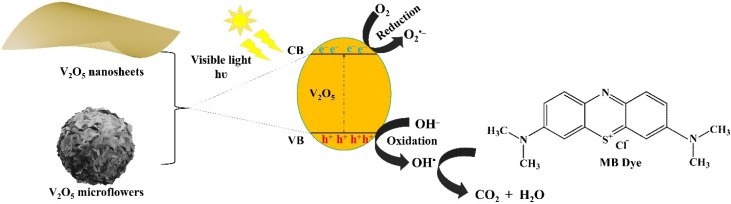
Photocatalytic degradation mechanism of MB by V_2_O_5_ nanosheets and microflowers under visible light illumination.

Based on well-documented literature and aforementioned results, the plausible photodegradation mechanism of MB by V_2_O_5_ nanosheets and microflowers under visible light illumination is proposed as shown in Fig. 12. Under visible light irradiation, V_2_O_5_ nanosheets become activated due to absorbing photons with incident energy surpassing the bandgap, consequently, generating electron–hole pairs. The excited electrons (e^–^) in the conduction band is instantly involved the reduction of oxygen molecules, resulting in the formation of superoxide (O_2_˙^–^) and/or hydroxide (OH^−^) free radicals in case of further reaction. The holes (h^+^) carrier occupied in the valent band simultaneously catalyze the production of hydroxyl radicals (OH˙) derived from adsorbed water at the surface of V_2_O_5_ nanosheets. The photogenerated species encompassing O_2_˙^–^, OH˙, and H_2_O_2_, instigate oxidative and reductive reactions, as a result, decomposing effectively methylene blue dye molecules absorbed on the nanosheet's surface.^50^

The comprehensively photocatalytic process results in degradation of the dye molecules into carbon dioxide (CO_2_) and water (H_2_O), demonstrating the efficacy of V_2_O_5_ nanosheets in degradation of environmental polluted substance through visible light-induced capability. A completed reaction diagram of this process is depicted pictorially in Equation (eqn (5)–(9)) and the Fig. 10:^51^

5 V_2_O_5_ + *hν* → V_2_O_5_ (h_VB_^+^ + e_cB_^−^)

6 h^+^ + H_2_O → OH˙ + H^+^

7 e^−^ + O_2_ → O_2_˙^−^

8 O_2_˙^−^ + H_2_O → H_2_O_2_ → 2OH˙

9 MB dye + O_2_˙^−^/OH˙ → the decomposed colorless

## Conclusions

In this study, we investigated the influence of surfactants on the morphology and photocatalytic performance of V_2_O_5_ materials synthesized *via* hydrothermal methods. The use of Pluronic P123 and CTAB surfactants led to the formation of V_2_O_5_ nanosheets and nanoflowers, respectively, with distinct morphological and structural properties. The V_2_O_5_-P123 nanosheets exhibited superior photocatalytic performance in the degradation of Methylene Blue (MB), achieving an efficiency of 96.72% after 150 minutes, which was attributed to their larger surface area and higher porosity compared to V_2_O_5_-CTAB. However, both materials showed limited degradation of Rhodamine B (RhB), emphasizing the impact of the pollutant's molecular structure on the photocatalytic activity. Material characterization through FE-SEM, TEM, XRD, and Raman spectroscopy confirmed the successful synthesis of high-purity V_2_O_5_ nanostructures with well-defined morphologies and crystal structures. The V_2_O_5_ nanosheets, with their high surface-to-volume ratio and porous nature, demonstrated enhanced photocatalytic properties, while the V_2_O_5_ microflowers, formed with CTAB, exhibited a more compact structure with lower porosity, affecting their catalytic efficiency. The results underscore the significant role that surfactants play in controlling the morphology and photocatalytic performance of V_2_O_5_ materials. These findings open up opportunities for the design of advanced photocatalysts with tailored properties for effective environmental remediation. Future work could focus on optimizing the synthesis conditions to enhance the photocatalytic degradation of various organic pollutants, including RhB, and exploring the potential for scale-up and real-world applications in wastewater treatment. Additionally, investigating the synergy between V_2_O_5_ and other nanomaterials, such as TiO_2_ or ZnO, may further improve the photocatalytic performance and stability of these materials.

## Data availability

Data for this article, including SEM, EDS, XRD, BET, FTIR, UV-vis, and photocatalytic performance are available at Open Science Framework at https://osf.io/a3thb/.

## Author contributions

NTMQ, DVL: investigation, data collection, writing – original draft preparation. DQD: resources, reviewing and editing; MT: writing – reviewing and editing. DL: visualization, editing, funding acquisition & supervision. All authors approved the manuscript.

## Conflicts of interest

The authors declare that they have no known competing financial interests or personal relationships that could have appeared to influence the work reported in this paper.

## Supplementary Material

RA-015-D5RA01050K-s001
