# Supplementary material for: Surfactant-tuned vanadium pentoxide for enhanced photocatalytic degradation of organic dyes: nanosheet vs. microflower morphologies
Source: RSC Adv. 2025 May 22;15(22):17277–89. doi: 10.1039/d5ra01050k (PMC12097202; doi:10.1039/d5ra01050k)
Supplement: RA-015-D5RA01050K-s001 [file RA-015-D5RA01050K-s001.pdf]

Supplementary Information (SI) for RSC Advances.
This journal is © The Royal Society of Chemistry 2025

Surfactant-Tuned Vanadium Pentoxide for Enhanced Photocatalytic Degradation of Organic dyes: Nanosheet vs. Microflower Morphologies

Nguyet Thi Minh Quan[1,+], Duy Van Lai [2,+], Matteo Tonezzer [3,4], Dat Quang Do[5,6], Duc D. La[7,8,*]

[1]*School of Engineering Physics, Hanoi University of Science and Technology (HUST), No 1 Dai Co Viet Street, Hanoi City, Vietnam.*

[2] *Institute of Materials Science, Vietnam Academy of Science and Technology, 18 Hoang Quoc Viet Street, Cau Giay District, Hanoi City, Vietnam.*

[3] *Research and Innovation Centre, Fondazione Edmund Mach, San Michele all' Adige, TN 38098, Italy*

[4] *Department of Chemical and Geological Sciences, University of Cagliari, Campus of Monserrato (CA), I09042, Monserrato, Italy*

[5]*School of Materials Science and Engineering (SMSE), Hanoi University of Science and Technology (HUST), No 1, Dai Co Viet Street, Hanoi, Vietnam*

[6]*Department of Natural Sciences, Hoa Lu University, Ninh Binh City, Vietnam*

[7]*Laboratory of Biophysics, Institute for Advanced Study in Technology, Ton Duc Thang University, Ho Chi Minh City, Vietnam,*

[8]*Faculty of Applied Sciences, Ton Duc Thang University, Ho Chi Minh City, Vietnam*

Corresponding authors: *laducduong@tdtu.edu.vn*

[+] These authors equally contributed

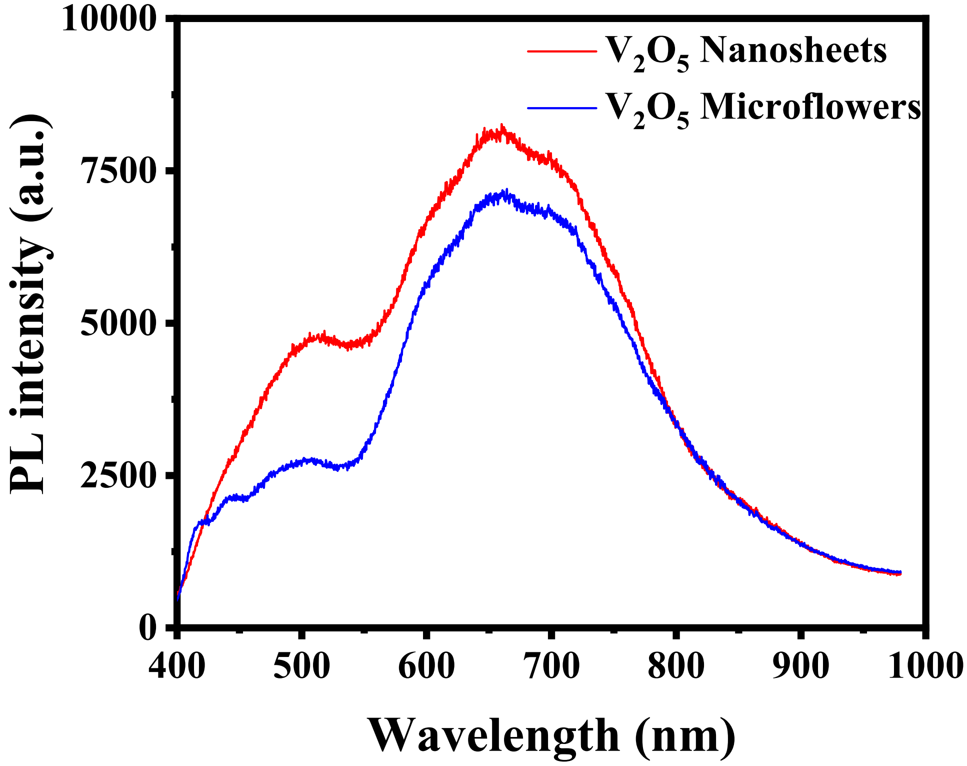

Figure S1. PL spectrum data of V_2O_5 nanosheets and microflowers at room temperature (excitation: 355 nm).

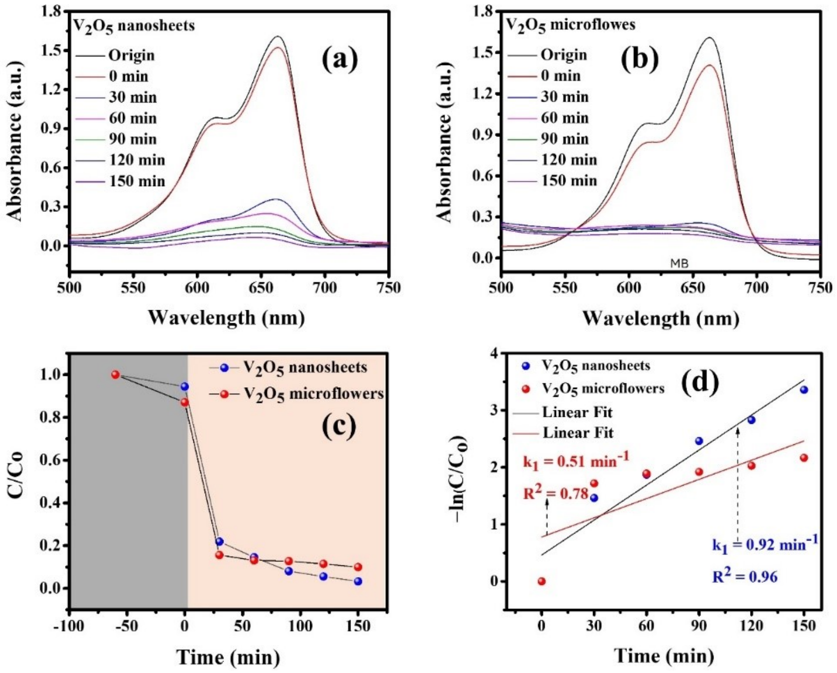

Figure S2. Photocatalytic degradation of Methylene Blue (MB) under simulated sunlight using V_2O_5 nanosheets and microflowers: (a, b) Absorbance spectra over irradiation time, (c) Photodegradation efficiency, and (d) $\ln(C/C_0)$ vs. irradiation time (150 minutes).

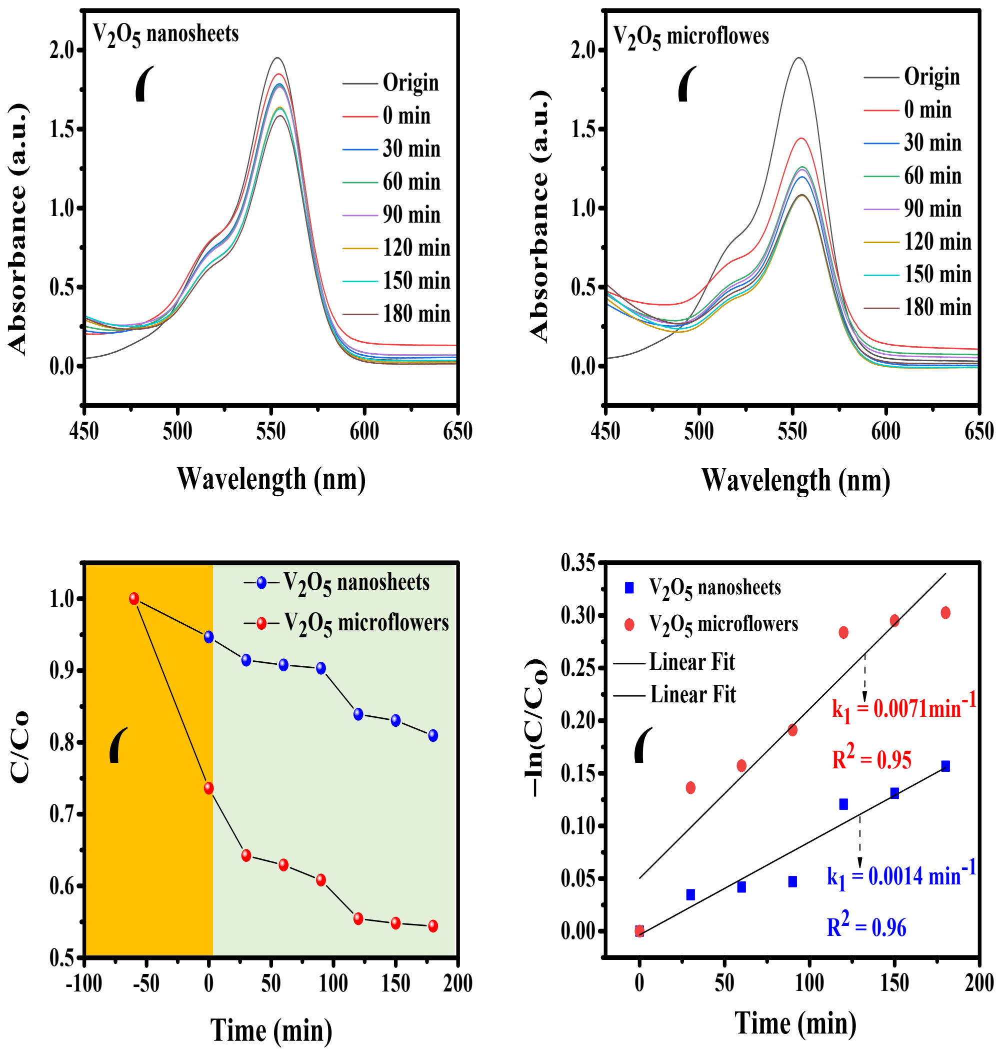

Figure S3. Photocatalytic degradation of Rhodamine B (RhB) under simulated sunlight using V_2O_5 nanosheets and microflowers: (a, b) Absorbance spectra over irradiation time, (c) Photodegradation efficiency, and (d) $\ln(C/C_0)$ vs. irradiation time.
